# Lumbar MRI-Based Deep Learning for Osteoporosis Prediction

**DOI:** 10.3390/diagnostics16030423

**Published:** 2026-02-01

**Authors:** Ue-Cheung Ho, Hsueh-Yi Lu, Lu-Ting Kuo

**Affiliations:** 1 Division of Neurosurgery, Department of Surgery, National Taiwan University Hospital, Taipei 100, Taiwan; coolive0510@hotmail.com; 2 Graduate Institute of Clinical Medicine, College of Medicine, National Taiwan University, Taipei 100, Taiwan; 3 Department of Industrial Engineering and Management, National Yunlin University of Science and Technology, Yunlin 640, Taiwan; hylu@yuntech.edu.tw; 4 Division of Neurosurgery, Department of Surgery, National Taiwan University Hospital, Yunlin Branch, Yunlin 640, Taiwan

**Keywords:** artificial intelligence, bone mineral density, deep learning, magnetic resonance imaging, osteoporosis

## Abstract

**Background**: Osteoporosis (OP) is characterized by reduced bone mineral density and increased fracture risk. Many spinal surgery patients have undiagnosed OP due to the lack of preoperative screening, leading to postoperative complications. Magnetic resonance imaging (MRI), a routine, non-invasive tool for spinal assessment, offers potential for opportunistic OP detection. This study aimed to develop deep learning models to identify OP using lumbar MRI. **Methods**: We retrospectively enrolled 218 patients (≥50 years) who underwent both lumbar MRI and dual-energy X-ray absorptiometry (DXA). After segmentation of vertebral bodies from T1- and T2-weighted MRI images, 738 images per sequence were extracted. Separate convolutional neural network (CNN) models were trained for each sequence. Model performance was evaluated using receiver operating characteristic curves and area under the curve (AUC). **Results**: Among tested classifiers, EfficientNet b4 showed the best performance. For the T1-weighted model, it achieved an AUC of 82%, with a sensitivity of 85% and specificity of 79%. For the T2-weighted model, the AUC was 83%, with a sensitivity of 86% and specificity of 80%. These results were superior to those of InceptionResNet v2 and ResNet-50 for both sequences. **Conclusions**: The AI models provided reliable OP classification without additional imaging or radiation. AI-based analysis of standard lumbar MRI sequences can accurately identify OP. These models may assist in early detection of undiagnosed OP in surgical candidates, enabling timely treatment and perioperative strategies to improve outcomes and reduce healthcare burden.

## 1. Introduction

Osteoporosis (OP) is a common yet frequently underdiagnosed metabolic bone disease characterized by reduced bone mineral density (BMD), increased bone fragility, and a higher risk of fractures [1]. In surgical populations—particularly those undergoing spinal procedures—undiagnosed OP can lead to impaired mobility, surgical complications, prolonged recovery, and even increased mortality [2]. With the general increase in global life expectancy, OP has become the most common metabolic bone disease worldwide and is associated with considerable public health problems and socioeconomic burdens [3]. Approximately 15% of men and 50% of postmenopausal women have OP. The prevalence of bone fractures among patients with OP is as high as 40% [4,5]. Individuals with OP show increased mortality rates compared with those without OP, particularly following the occurrence of vertebral or non-vertebral fractures [2,3]. The one-year mortality rate in patients with OP ranges from 17.1% to 33%, depending on factors such as age, sex, ethnicity, and fracture site [6,7].

OP is associated with various factors, including sex, age, family history, hysterectomy, low body mass index, physical inactivity, inadequate calcium and vitamin D intake, excessive alcohol consumption, smoking, and endocrine and cardiometabolic factors. To reduce the incidence and poor outcomes of OP, it is essential to clarify the risk factors of OP, increase awareness of asymptomatic OP, and implement appropriate early detection and management measures. However, despite the importance of identifying individuals with an increased risk for OP, routine screening for OP is not performed extensively because OP is usually diagnosed only after the occurrence of a fracture. This lack of routine OP screening can be attributed to the limited availability of dual-energy X-ray absorptiometry (DXA) machines, concerns regarding radiation exposure, and the reimbursement policies of national and private insurance entities. Given that these factors hinder the broader implementation of proactive OP screening efforts, the development of strategies to overcome these challenges and enhance OP prevention measures is essential.

The prevalence of OP among individuals aged over 50 years who have undergone spinal surgery ranges from 14.5% to 19.9% in men and 43.0% to 52.8% in women, which is higher than the prevalence of OP in the general population [8,9]. Several studies have suggested that OP in elderly patients undergoing spinal surgery is associated with an increased risk of complications, such as screw loosening, adjacent segment fractures, proximal junctional kyphosis, and decreased fusion rates [9,10]. The limited availability of DXA and the frequent preoperative underdiagnosis of OP increase the risk of postoperative complications in patients undergoing spinal surgery.

In recent years, interest has grown in alternative imaging modalities for assessing BMD. Quantitative computed tomography (CT) can assess volumetric BMD and is especially useful for detecting trabecular bone loss. Magnetic resonance imaging (MRI) can assess bone microarchitecture and bone marrow composition. However, their accuracy and clinical utility in BMD assessment require further validation. In contrast to CT, which is associated with a risk of radiation exposure and is not obligatory for preoperative planning, MRI is more commonly used because of its established role as a standard imaging technique for evaluating the severity of intervertebral disc degeneration, nerve root compression, or spinal stenosis in patients with neurological symptoms. However, relatively few studies have explored the use of MRI for assessing BMD, and the existing literature is notably heterogeneous due to variations in study design, imaging methodologies, and MRI protocols. In recent years, artificial intelligence (AI) has advanced rapidly in medical research, particularly in the field of image recognition. Its integration into medical imaging has enabled more accurate assessments, reduced physician workload, minimized diagnostic errors, and enhanced disease prediction and detection. Therefore, this study aimed to evaluate the predictive ability of lumbar spine MRI for OP using a deep learning neural network and conventional T1-weighted and T2-weighted MRI images. This study is intended as a single-center feasibility investigation evaluating MRI-based deep learning models using routine T1- and T2-weighted lumbar MRI for opportunistic osteoporosis screening, with dual-energy X-ray absorptiometry as the reference standard.

## 2. Methods

This was a retrospective study, and its primary objective was to analyze the efficacy of a convolutional neural network (CNN) algorithm in classifying spinal MRI images for the prediction of OP. Therefore, we established a CNN model to predict the risk of OP using spinal MRI images. This study was approved by the Institutional Review Board (IRB) of the National Taiwan University Hospital (IRB number: 202112143RINA), and the requirement for informed consent was waived due to the retrospective nature of the study.

### 2.1. MRI Dataset

A total of 218 adult patients (both men and women aged ≥ 50 years) who underwent both lumbar MRI and DXA at four lumbar levels between 2015 and 2021 at National Taiwan University Hospital and its Yunlin Branch were included in this study.

Patients were excluded if they were under 50 years of age, had a history of lumbar spine surgery or instrumentation, had experienced trauma, or had spinal tumors, compression fractures, or known inflammatory or infectious spinal conditions.

To avoid the influence of secondary OP, patients suspected of having systemic diseases or long-term medication use affecting bone metabolism were further assessed. Secondary OP was defined as low bone mass with deteriorated bone microarchitecture caused by underlying medical conditions or medications. Suspicion was based on clinical history, comorbidities, physical examination findings, or medication profiles. Further laboratory evaluation was arranged at the discretion of the treating surgeon and included, when necessary, tests such as C-reactive protein, erythrocyte sedimentation rate, serum calcium, phosphate, alkaline phosphatase, liver enzymes, creatinine, 25-hydroxyvitamin D, cortisol, adrenocorticotropic hormone (ACTH), and thyroid function. Patients confirmed to have secondary OP were excluded from the study. Lumbar DXA provided vertebra-specific bone mineral density values and corresponding T-scores for L1–L4. Each segmented vertebral MRI image was matched to its corresponding vertebra-specific DXA measurement, allowing anatomically consistent vertebra-level supervision during model training. To ensure accurate vertebra-level correspondence between DXA and MRI, vertebrae with severe degenerative changes, deformity, or compression fractures were excluded from the analysis. As a result, each vertebral MRI sample was labeled using the DXA measurement obtained from the same vertebral level, and femoral neck or total hip DXA measurements were not used for osteoporosis classification in this study.

**Figure 1:**
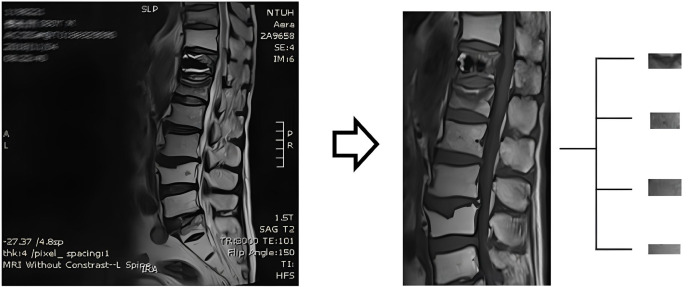
Image pre-processing and segmentation.

All patients underwent 3T Magnetom Verio MRI systems (Siemens Healthcare, Erlangen, Germany) and T1-weighted and T2-weighted MRI images were used in this study. The sagittal midline image of a single vertebra was segmented from an MRI image as a sample in the dataset. Four vertebrae (first to fourth) of the lumbar spine, which were comparatively evaluated to determine their BMD, were segmented (Figure 1). However, vertebrae with compression fractures that could potentially introduce bias to the BMD calculation were excluded. A total of 738 T1-weighted and 738 T2-weighted images of vertebrae generated from 218 patients were collated for analysis. T1-weighted and T2-weighted images were used separately to generate different prediction models.

To maintain consistency with the information in previous studies and clinical practice guidelines, we defined OP as a T-score of 2.5 standard deviations below the standard value for young people measured using DXA. The presence and absence of OP were determined using DXA according to this definition. Image processing included optimizing the visual effects of the MRI images using a high-pass filter, removing noise to enlarge the images, and creating models to categorize the vertebral images as ‘OP’ or ‘non-OP’.

### 2.2. Data Pre-Processing

In this phase, we modified the MRI images into more optimal representations of a pre-trained input before they could be processed using CNN techniques to improve model performance. The process included data cleaning by removing noise from the images to retain the entire lumbar spine in each image. Thereafter, data segmentation was performed to extract relevant samples by dividing each lumbar spine image into multiple vertebral segments, each of which corresponded to a different trained sample. Each vertebral sample was defined as a two-dimensional mid-sagittal image of an individual vertebral body (L1–L4). The region of interest encompassed the vertebral body including the cortical shell and internal marrow space, while intervertebral discs, posterior elements, and surrounding soft tissues were excluded. Segmentation was performed using a standardized manual crop-based approach rather than automated or pixel-level mask segmentation, following predefined anatomical criteria. Each vertebral sample was rescaled to 224 × 224 pixels to fit the ResNet architecture as input images in the CNN model. Vertebral samples with compression fractures were excluded from the dataset because fracture images may lead to inaccurate bone density predictions.

All images of vertebrae were pre-processed through filtering, labeling, extremum removal, grayscale conversion, and data enhancement before being input into the CNN model. We used a Laplacian filter with a second-order derivative function, which is a type of high-pass filter, to optimize visual effects and sharpen the images [11]. The MRI images were classified into three dimensions and included redundant data such as case number and the time and location where the procedure was performed. Utilizing a noise removal and grayscale process enabled the model to focus on the area of interest from a two-dimensional perspective and could reduce the number of subsequent model calculations. Minor modifications were made to the vertebral images by rotating and transposing the matrices of the mirror images to increase the number of training samples and to reduce overfitting in the prediction model. Data augmentation was applied only to the training dataset after data splitting and was not used for validation or testing datasets. After data augmentation, 1520 OP and 5860 non-OP images were generated from a total of 7380 images.

### 2.3. CNN Model

**Figure 2:**
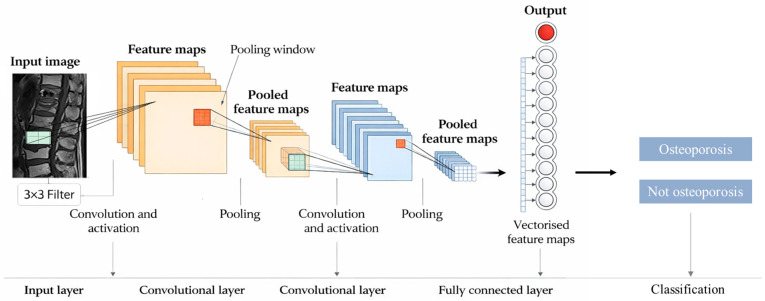
Convolutional neural network architecture.

We used a CNN with fully connected layers to extract the features of the vertebral images. The architecture of the CNN is shown in Figure 2. Peak signal-to-noise ratio was used to optimize the visual effects of each image. Confusion matrix, random seed, and K-fold validation were used to evaluate the performance of the classifiers.

The vertebral image set was classified using EfficientNet b4, which employs a simple and efficient compound coefficient with a scaling method to expand the CNN [12]. Unlike traditional methods, each dimension is uniformly scaled using a fixed set of scaling factors to develop a series of EfficientNet models. EfficientNet b4 uses a FLOPS similar to that utilized by the widely used ResNet-50 while improving top-1 accuracy from 76.3% of ResNet-50 to 82.6% (+6.3%) [12]. A convolution kernel, also known as a filter, is a matrix that extracts certain features from an input image. The number of kernels controls the number of feature maps and determines the receptive field, which represents the size of the area of the original image receptive to neurons at different positions in the network. Stride is one of the methods used by CNN to control the lengths and widths of feature images. The length and width of the images were set to be equal. Padding was used to complement pixels with a value of zero around the feature image. The convolution layer was created by sliding the image through different kernels with feature-receptive fields projected onto an element in the new feature image. Max pooling with a 2 × 2 window was applied to extract the maximum value from the feature map. The fully connected layer is mainly used as a classifier for final feature extraction.

The image-based feature extraction was performed using EfficientNet-B4 as the backbone convolutional neural network. The model was initialized with ImageNet-pretrained weights rather than being trained from scratch, in order to improve convergence and reduce overfitting given the limited size of medical imaging data. Input CT images were resized to 380 × 380 pixels, consistent with the original EfficientNet-B4 architecture specification. During training, a batch size of 16 was used. Model optimization was carried out using the Adam optimizer with an initial learning rate of 1 × 10^−4^. A ReduceLROnPlateau learning rate scheduler was applied, which reduced the learning rate by a factor of 0.5 if the validation loss did not improve for 5 consecutive epochs. The network was trained for a maximum of 100 epochs. To prevent overfitting, early stopping was implemented based on validation loss, with training terminated if no improvement was observed for 10 consecutive epochs. The model checkpoint achieving the lowest validation loss was selected for final evaluation and testing.

### 2.4. Performance Evaluation

The receiver operating characteristic (ROC) curve is a commonly used performance index for binary classifiers. Sensitivity and specificity are used to establish the ROC curve. A curve skewed towards the coordinate (0,1) means the prediction is more accurate. The area under the curve (AUC) is the area beneath the ROC curve. AUC was used to evaluate the quality of the models. The values range from 0 to 1, and the closer the value is to 1, the better the performance of the model and vice versa. Direct statistical comparison between T1- and T2-based AUCs was not performed, as the models were trained independently on different MRI sequences and were not intended for head-to-head comparison.

### 2.5. Data Availability

The datasets generated during and/or analyzed during the current study are available from the corresponding author upon reasonable request.

## 3. Results

A total of 218 patients were included in the final analysis, comprising 48 men (22.0%) and 170 women (78.0%). The mean age of the study population was 72.69 years, with a standard deviation of 9.96 years. This cohort reflects a typical older adult population undergoing bone mineral density assessment, with a predominance of postmenopausal women.

After pre-processing, the dataset was randomly divided into two in an 80:20 ratio. Training and validation of the CNN model were performed using 80% of the images, whereas the final testing was performed using 20% of the images. The K-fold cross-validation method was used to train and validate the model. The vertebral MRI samples were randomly partitioned into K datasets. K−1 datasets were used for training, and one dataset was used for validation. The advantage of this method is that it repeatedly uses randomly generated sub-datasets for training and validation [13]. The proposed models were developed using a Python 3.7 environment. The original dataset comprised 1476 vertebral images (304 OP and 1172 non-OP images). After image augmentation, the dataset was expanded to 7380 images (1520 OP and 5860 non-OPs). Different models were developed using T1-weighted and T2-weighted MRI images. To develop an optimal classification model, different models were developed and evaluated using three classifiers: EfficientNet b4, InceptionResNet v2, and ResNet-50. To determine the most accurate model for osteoporosis diagnosis, 80% of the preselected samples were used for five-fold cross-validation (K = 5), and the remaining 20% were reserved for independent testing. For the augmented dataset, EfficientNet-b4 demonstrated the highest diagnostic performance among the tested models. The AUC was 0.82 (95% CI, 0.74–0.90) for the T1-weighted model and 0.83 (95% CI, 0.75–0.91) for the T2-weighted model.

**Figure 3:**
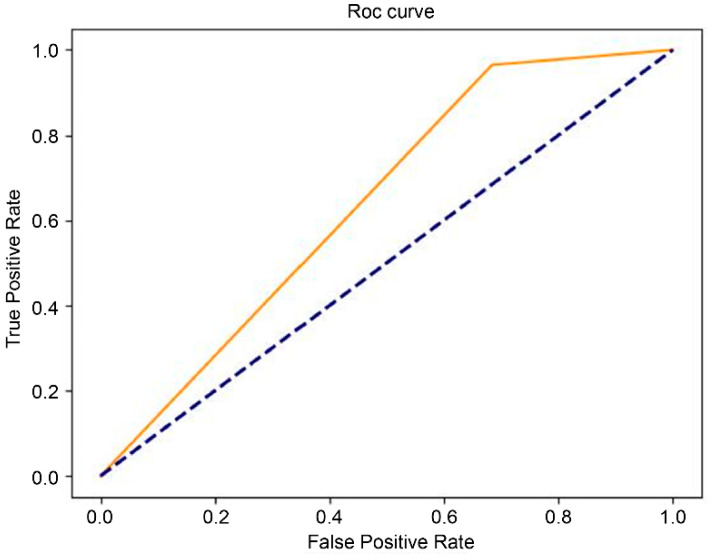
The ROC curve of EfficientNet b4 in the T1 model (AUC = 0.56), compared with chance level.

**Figure 4:**
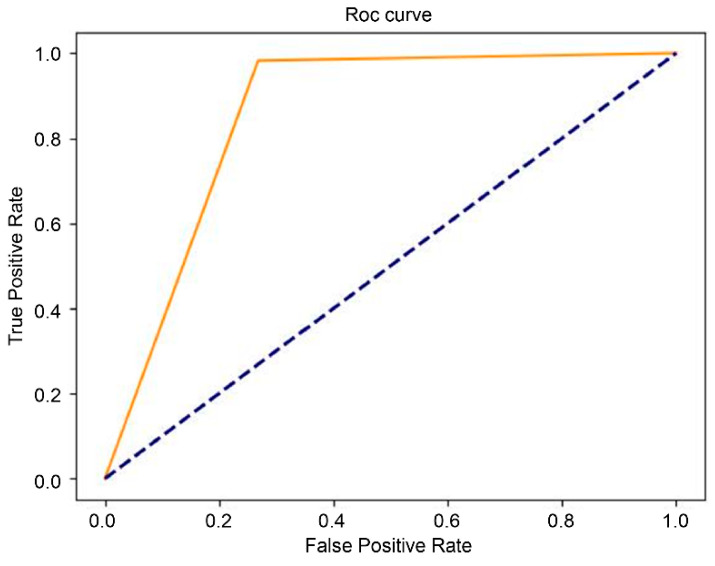
The ROC curve of EfficientNet b4 in the augmented T1 model  (AUC = 0.82), compared with chance level.

**Figure 5:**
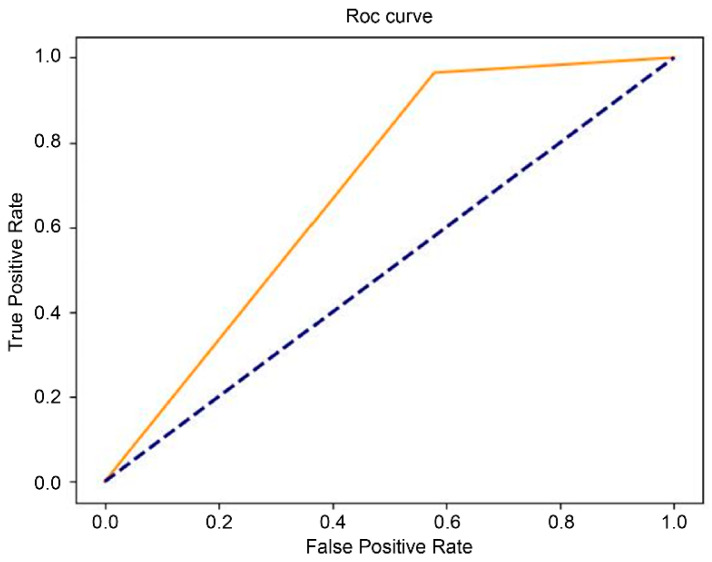
The ROC curve of EfficientNet b4 in the T2 model  (AUC = 0.59), compared with chance level.

**Figure 6:**
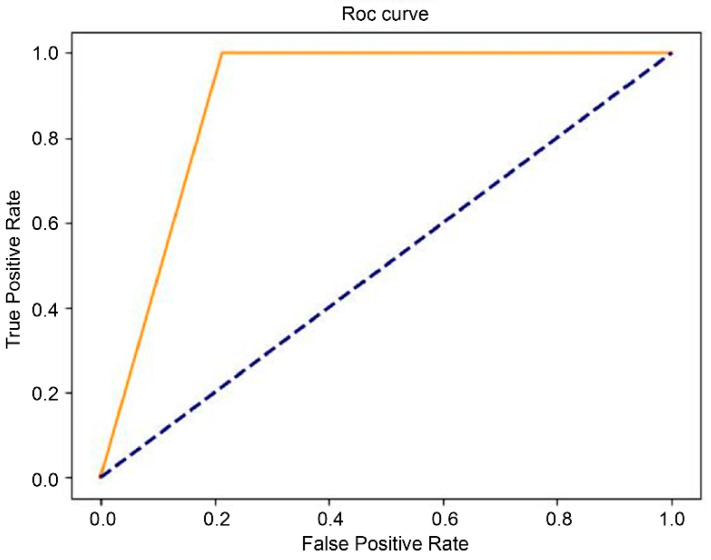
The ROC curve of EfficientNet b4 in the augmented T2 model  (AUC = 0.83), compared with chance level.

The results of the final testing of the T1-weighted and T2-weighted models using the original dataset showed that the model with the EfficientNet b4 classifier outperformed InceptionResNet v2 and ResNet-50 in terms of the performance measures (Table 1 and Table 2). We further investigated the performance of the augmented dataset, and the results showed that EfficientNet b4 had the best AUC value: 82% for the T1 model (Table 1) and 83% for the T2 model (Table 2). The ROC curves of the EfficientNet b4 classifier in the T1 and T2 models based on the original and augmented datasets are shown in Figure 3, Figure 4, Figure 5 and Figure 6.

## 4. Discussion

DXA is the gold standard for OP diagnosis. The definition of OP proposed by the World Health Organization is based on the T-score of the femoral neck or lumbar spine measured using DXA and is defined as a T-score of 2.5 standard deviations or more below the mean for young female adults. Although there are certain limitations associated with BMD assessment using DXA, including the effect of alterations in body composition on BMD measurement and potential diagnostic bias from degenerative spines due to the two-dimensional imaging technique [14], these limitations should not discourage its use. BMD is a pivotal intervention threshold in fracture risk assessment algorithms because diminished bone density substantially increases an individual’s susceptibility to fragility fractures (https://www.sheffield.ac.uk/FRAX/ (accessed on 30 July 2025)). Approximately 50% of women and 20% of men will have an osteoporotic fracture in their lifetime [6]. Unfortunately, osteoporotic fractures are frequently asymptomatic and underdiagnosed because OP is usually diagnosed after the occurrence of a symptomatic fracture. In addition, some fragility fractures occur in individuals whose BMD values do not initially meet the diagnostic thresholds for OP [4]. Consequently, the initiation of treatment may be substantially delayed, which is problematic given that the patients have an increased risk of future fractures.

Vertebral fractures are the most prevalent fractures in patients with OP [3]. A systematic review has indicated that patients with OP are twice as likely as those with normal bone quality to experience screw loosening following spinal fusion surgery [10]. Given that bone quality assessment is not obligatory in the preoperative planning of spine surgery, and considering the challenges in extensively implementing the use of DXA for OP screening, exploration of alternative methods for preoperative bone quality evaluation is imperative for mitigating the occurrence of postoperative morbidities after spine surgery.

There is growing evidence of the application of alternative imaging modalities, such as CT and MRI, for the assessment of BMD. CT has been utilized in several studies to predict BMD as assessed using DXA. However, the diagnostic accuracy of these modalities for OP has exhibited considerable variability in previous studies, with AUCs ranging from 0.74 to 0.97, owing to differences in the diagnostic thresholds and the anatomical regions of interest utilized in these previous studies [15,16,17]. Compared to DXA and CT, quantitative computed tomography (qCT) offers distinct advantages in the precise assessment of three-dimensional anatomic localization and provision of a direct measurement of density [18]. Although several studies have been conducted to compare qCT and DXA for the evaluation of BMD in the vertebral column [19,20], there is no consensus on the classification of diagnostic categories based on measurements obtained from spine qCT. Moreover, the use of CT for predicting BMD is hindered by factors such as radiation exposure and the fact that CT is not a mandatory preoperative assessment for patients scheduled for spinal surgery. In contrast, MRI is more commonly used because it is well-established as a standard imaging modality for the preoperative evaluation of patients with neurological symptoms. In addition, MRI has advantages over CT because it does not use ionizing radiation, provides high-resolution images, and can evaluate additional physiological properties of bone beyond its structural aspects [21]. Several MRI parameters, including vertebral bone quality (VBQ) score [22], MRI-based score (M-score) [23], and MRI-derived quantitative perfusion markers [24], have been used for assessment of bone quality in the spine. All these parameters are acquired using standard diagnostic MRI sequences and show promising correlations with BMD. There are some additional MRI protocols designed for the assessment of different physiological properties of the spine, including magnetic resonance spectroscopy [25,26] and chemical shift-encoding-based water–fat MRI [27] for evaluating the nonmineralized bone compartment through the extraction of the bone marrow fat fraction and proton density fat fraction; ultrashort echo time imaging of cortical bone to evaluate its quantity and quality [28]; and quantitative susceptibility mapping for assessment of trabecular bone microstructure [29]. While previous MRI techniques primarily utilized the signal originating from the bone marrow to visualize the trabecular microstructure, recent advanced MRI postprocessing techniques allow for more direct imaging of the bone tissue. These techniques have the potential to enhance fracture risk assessment in patients with OP; however, they have not yet been introduced into clinical practice. Furthermore, the availability of the required equipment is limited, and they have only been used in research settings.

Deep learning for image recognition in medical research has developed rapidly in recent years, significantly facilitating the identification and diagnosis of various diseases, such as cancer, cerebrovascular diseases, and cardiovascular diseases, by recognizing characteristic features in images [30]. The integration of deep learning into medical image assessment has led to accurate evaluations, reduced physician workload, reduced diagnostic errors, and enhanced disease prediction and detection. Owing to its adeptness in model learning and automated extraction of target features, the CNN algorithm, which is rooted in deep learning principles, is progressively taking over the role of supervised learning in the field of computer-aided medical image processing in AI [31]. In the present study, the EfficientNet b4 algorithm, which is based on deep learning, was used to predict OP based on MRI image characteristics. The AUC of EfficientNet b4 indicated that the algorithm demonstrated superior performance in predicting OP compared with EfficientNet v2 and ResNet-50. Moreover, the augmented MRI image models showed significant improvement. The models developed in this study exhibited good performance without preliminary image feature engineering and remained effective across diverse imaging scanners, with the potential for use by non-experts. These findings align with the conclusions drawn from previous research [32,33], underscoring the efficacy of deep learning-based MRI image algorithms in significantly enhancing image quality, streamlining the identification of disease-related features, and ultimately enhancing diagnostic efficiency. Notably, one advantage of our approach to the prediction of OP over those used in previous studies is that most previous studies were focused on classification using demographic, clinical, biochemical, genetic, nutrient, or lifestyle data [34,35], whereas we used MRI images, which are safer and more convenient in terms of patient privacy and data collection in practical applications. To the best of our knowledge, the present study is the first to use exclusively MRI-based AI methods to predict OP in patients undergoing spinal surgery.

Bone marrow adipocytes, an essential component of the bone marrow microenvironment, interact with other cell types in the marrow and regulate bone remodeling. The development of marrow fat in humans is age-dependent, and marrow adiposity increases with age-related bone loss [36,37]. Fat in the marrow is traditionally believed to replace the space left by trabecular bone loss in the elderly and patients with OP [38]. Furthermore, studies have demonstrated increased infiltration of marrow adipocytes in individuals with OP compared to their age-matched individuals without OP [39]. However, it has been suggested that there is no correlation between peripheral fat and bone marrow adiposity [40]. Estrogen deficiency during menopause contributes to the development of OP. The decline in estrogen levels following menopause not only disrupts the coupling of bone remodeling units but also leads to an increase in marrow adiposity accompanied by a reduction in bone mass [37,41].

MRI is the most common imaging modality used for the evaluation of patients with spinal disease and offers excellent soft tissue resolution and distinct contrast between fat and water. In modern clinical MRI, notable differences in T1 and T2 relaxation have facilitated qualitative interpretations of fat and water constituents within tissues and organs. This distinctive capability of MRI has been used to investigate and quantitatively assess muscle atrophy and adipose tissue infiltration [42]. Various MRI-derived metrics based on these characteristics, such as the VBQ and M-scores, have been developed for the evaluation of bone quality using T1-weighted MRI images. T2-weighted MRI has also been used in the assessment of OP [43,44] owing to its ability to detect inhomogeneities arising from susceptibility differences at the interface between the bone marrow and trabecular bone. T2-weighted MRI has also been used to evaluate the remodeled trabecular microstructure. In addition, although T1-weighted MRI provides strong and stable contrast for fat-rich marrow, osteoporosis-related changes involve not only increased marrow fat content but also alterations in trabecular microstructure and marrow microenvironment. T2-weighted imaging is influenced by both fat and water components and may therefore better capture heterogeneity in marrow composition and microenvironmental changes associated with trabecular deterioration, which may contribute to the slightly higher diagnostic performance observed for the T2-weighted model in the present study. Pathological changes in OP manifest as changes detected on T1-weighted and T2-weighted MRI. Considering the extensive rapid data acquisition, model learning, and automated feature extraction capabilities of AI and the unique characteristics of T1- and T2-weighted sequences, incorporating AI into the utilization of T1-weighted or T2-weighted sequences has the potential to significantly enhance diagnostic accuracy. The results of this study demonstrate the solid diagnostic performance of our algorithm, with an AUC of 82% for the T1-weighted model and 83% for the T2-weighted model. While both models performed similarly, the slightly higher AUC of the T2-weighted model suggests a potential advantage and highlights its value in assessing bone quality. These findings may encourage further investigation into the optimal MRI sequences for evaluating bone quality.

Advanced 2D and 3D quantitative segmentation algorithms enable the measurement of adipose content and proportion within muscle volumes or areas [45]. Some radiology subspecialties include the investigation of texture analysis, which is an advanced method of image pattern analysis with significant implications [46]. As the use of AI in MRI continues to progress, various algorithms can offer more accurate predictions of spinal constituents. This advancement holds the potential for broader applications in the diagnosis of spinal metabolic diseases characterized by distinct changes in components.

AI algorithms have been used in previous studies to integrate a diverse range of clinical features into a comprehensive model for predicting and distinguishing individuals susceptible to OP [34,47,48,49]. In general, both excessive reduction and augmentation of features for deep learning lead to poor performance [50]. In the present study, we constructed the prediction model using a single MRI factor, and that yielded a comparatively favorable AUC of 83%. MRI is a viable option for quantitative, radiation-free evaluation of osteoporotic bone and is rapidly evolving owing to the variety of its sequences and methodologies available. Our findings present a novel simplified approach that allows for more efficient prediction of bone quality. Our findings have the potential to facilitate preoperative assessment of OP in patients with spinal diseases, pending the development of additional imaging modalities or AI-based algorithms for more precise prediction of bone quality. In addition, our findings could facilitate the timely initiation of OP treatment and optimize perioperative management, including the use of anabolic agents such as teriparatide, cement augmentation to enhance screw fixation, and individualized postoperative rehabilitation, thereby mitigating the risk of postoperative complications.

Recent studies have reported higher diagnostic performance for CT-based opportunistic osteoporosis screening, particularly in recent deep learning-based approaches [15,16,17]. However, CT- and MRI-based approaches provide complementary rather than competing information. CT-based methods primarily reflect bone mineral density and attenuation-related measures, whereas MRI is sensitive to bone marrow composition and microenvironmental changes related to bone quality. Importantly, unlike CT, MRI does not involve ionizing radiation and is routinely used for the evaluation of degenerative spinal diseases in clinical practice. Given that lumbar MRI is frequently obtained in this patient population, MRI-based opportunistic screening may offer added clinical value as a safe and readily available complementary approach rather than a replacement for CT-based methods.

This study is subject to certain limitations that warrant acknowledgment. Because imaging was performed using a single 3T MRI system at one institution, the present results should be interpreted within this single-center setting. The current analysis focused on discrimination performance using cross-validation and an internal hold-out test set. Threshold optimization, cost-sensitive learning strategies, and threshold-dependent performance metrics, including precision–recall, F1 score, positive predictive value, and negative predictive value, were not evaluated, as this study focused on vertebra-level discrimination rather than optimization of clinically defined decision thresholds. Calibration assessment and decision-curve analysis were not performed, as the primary objective of this study was feasibility evaluation rather than clinical decision modeling. Firstly, the data for this study were retrospectively collected from the National Taiwan University Hospital and its Yunlin Branch, emphasizing the importance of a larger sample size involving multiple institutions to ensure the generalizability of our findings. Secondly, vertebral MRI samples were split at the image level rather than strictly at the patient level. Although this approach maximized sample utilization for feasibility evaluation, it may introduce correlation between samples derived from the same individual. In addition, patient-level aggregation strategies, such as probability pooling across multiple lumbar vertebrae (L1–L4), were not applied, as the primary objective of this study was vertebra-level model evaluation. These approaches require predefined patient-level decision rules and outcome-driven validation and will be addressed in future prospective studies with external validation cohorts. Thirdly, our analysis exclusively relied on T1-weighted and T2-weighted MRI. Incorporating a broader range of MRI sequences, made possible by contemporary MRI technology, has the potential to enhance predictive accuracy. Finally, it should be noted that our study did not include an assessment of patients with compression fractures or those with inflammatory or infectious diseases. It is important to acknowledge that some patients may have coexisting degenerative spine diseases. These limitations highlight the need for future large-scale, multicenter studies to validate and extend our findings across diverse populations and clinical settings.

In conclusion, the results of this study demonstrated that AI models exhibit high accuracy and robust performance in identifying OP using routine spinal MRI. Incorporating these algorithms into clinical practice could assist healthcare providers in recognizing previously undiagnosed OP and enable the timely initiation of appropriate perioperative interventions. This approach has the potential to enhance surgical outcomes, reduce postoperative complications, and ultimately alleviate the burden on healthcare systems.

## Figures and Tables

**Table 1:** Results obtained using T1-weighted MRI images.

Models	Sensitivity	Specificity	AUC
Original dataset			
EfficientNet b4	67%	81%	56%
InceptionResNet v2	61%	85%	65%
ResNet-50	48%	89%	66%
With augmentation			
EfficientNet b4	85%	79%	82%
IceptionResNet v2	77%	83%	80%
ResNet-50	84%	71%	78%

AUC, area under the curve.

**Table 2:** Results obtained using T2-weighted MRI images.

Models	Sensitivity	Specificity	AUC
Original dataset			
EfficientNet b4	67%	83%	59%
InceptionResNet v2	58%	85%	65%
ResNet-50	60%	82%	58%
With augmentation			
EfficientNet b4	86%	80%	83%
IceptionResNet v2	80%	83%	80%
ResNet-50	80%	78%	79%

AUC, area under the curve.

## Data Availability

The datasets generated during and/or analyzed during the current study are available from the corresponding author upon reasonable request.

## References

[1] Reid I.R., Billington E.O. (2022). Drug therapy for osteoporosis in older adults. Lancet.

[2] Nazrun A.S., Tzar M.N., Mokhtar S.A., Mohamed I.N. (2014). A systematic review of the outcomes of osteoporotic fracture patients after hospital discharge: Morbidity, subsequent fractures, and mortality. Ther. Clin. Risk Manag..

[3] Compston J.E., McClung M.R., Leslie W.D. (2019). Osteoporosis. Lancet.

[4] Clynes M.A., Harvey N.C., Curtis E.M., Fuggle N.R., Dennison E.M., Cooper C. (2020). The epidemiology of osteoporosis. Br. Med. Bull..

[5] Reid I.R. (2020). A broader strategy for osteoporosis interventions. Nat. Rev. Endocrinol..

[6] Glinkowski W., Narloch J., Krasuski K., Śliwczyński A. (2019). The increase of osteoporotic hip fractures and associated one-year mortality in Poland: 2008–2015. J. Clin. Med..

[7] Guzon-Illescas O., Perez Fernandez E., Crespí Villarias N., Quirós Donate F.J., Peña M., Alonso-Blas C., García-Vadillo A., Mazzucchelli R. (2019). Mortality after osteoporotic hip fracture: Incidence, trends, and associated factors. J. Orthop. Surg. Res..

[8] Chin D.K., Park J.Y., Yoon Y.S., Kuh S.U., Jin B.H., Kim K.S., Cho Y.E. (2007). Prevalence of osteoporosis in patients requiring spine surgery: Incidence and significance of osteoporosis in spine disease. Osteoporos. Int..

[9] Fan Z.Q., Yan X.A., Li B.F., Shen E., Xu X., Wang H., Zhuang Y. (2023). Prevalence of osteoporosis in spinal surgery patients older than 50 years: A systematic review and meta-analysis. PLoS ONE.

[10] Ogiri M., Nishida K., Park H., Rossi A. (2023). Systematic literature review and meta-analysis on the clinical outcomes of spine surgeries in patients with concurrent osteoporosis. Spine Surg. Relat. Res..

[11] Makandar A., Halalli B. (2015). Image enhancement techniques using highpass and lowpass filters. Int. J. Comput. Appl..

[12] Tan M., Le Q.V. (2019). EfficientNet: Rethinking model scaling for convolutional neural networks. International Conference of Machine Learning.

[13] Wong T.-T., Yeh P.-Y. (2020). Reliable accuracy estimates from k–fold cross validation. IEEE Trans. Knowl. Data Eng..

[14] Golding P.H. (2022). Dual-energy x-ray absorptiometry (DXA) to measure bone mineral density (BMD) for diagnosis of osteoporosis—Experimental data from artificial vertebrae confirms significant dependence on bone size. Bone Rep..

[15] Buckens C.F., Dijkhuis G., de Keizer B., Verhaar H.J., de Jong P.A. (2015). Opportunistic screening for osteoporosis on routine computed tomography? An external validation study. Eur. Radiol..

[16] Lee S.Y., Kwon S.S., Kim H.S., Yoo J.H., Kim J., Kim J.Y., Min B.C., Moon S.J., Sung K.H. (2015). Reliability and validity of lower extremity computed tomography as a screening tool for osteoporosis. Osteoporos. Int..

[17] Pickhardt P.J., Lauder T., Pooler B.D., Muñoz Del Rio A., Rosas H., Bruce R.J., Binkley N. (2016). Effect of IV contrast on lumbar trabecular attenuation at routine abdominal CT: Correlation with DXA and implications for opportunistic osteoporosis screening. Osteoporos. Int..

[18] Genant H.K., Block J.E., Steiger P., Glueer C.C., Smith R. (1987). Quantitative computed tomography in assessment of osteoporosis. Semin. Nucl. Med..

[19] Kim K., Song S.H., Kim I.J., Jeon Y.K. (2021). Is dual-energy absorptiometry accurate in the assessment of bone status of patients with chronic kidney disease?. Osteoporos. Int..

[20] Kinsella S., Murphy K., Breen M., O’Neill S., McLaughlin P., Coyle J., Bogue C., O’Neill F., Moore N., McGarrigle A. (2015). Comparison of single CT scan assessment of bone mineral density, vascular calcification and fat mass with standard clinical measurements in renal transplant subjects: The ABC HeART study. BMC Nephrol..

[21] Martel D., Monga A., Chang G. (2022). Osteoporosis imaging. Radiol. Clin. N. Am..

[22] Aynaszyan S., Devia L.G., Udoeyo I.F., Badve S.A., DelSole E.M. (2022). Patient physiology influences the MRI-based vertebral bone quality score. Spine J..

[23] Bandirali M., Di Leo G., Papini G.D.E., Messina C., Sconfienza L.M., Ulivieri F.M., Sardanelli F. (2015). A new diagnostic score to detect osteoporosis in patients undergoing lumbar spine MRI. Eur. Radiol..

[24] Huang Z., Lin Q., Wang J., Zhan Z., Tu X. (2019). Relationship between quantitative parameters of lumbar vertebral perfusion and bone mineral density (BMD) in postmenopausal women. Adv. Clin. Exp. Med..

[25] Li X., Kuo D., Schafer A.L., Porzig A., Link T.M., Black D., Schwartz A.V. (2011). Quantification of vertebral bone marrow fat content using 3 Tesla MR spectroscopy: Reproducibility, vertebral variation, and applications in osteoporosis. J. Magn. Reson. Imaging.

[26] Yeung D.K.W., Griffith J.F., Antonio G.E., Lee F.K.H., Woo J., Leung P.C. (2005). Osteoporosis is associated with increased marrow fat content and decreased marrow fat unsaturation: A proton MR spectroscopy study. J. Magn. Reson. Imaging.

[27] Burian E., Subburaj K., Mookiah M.R.K., Rohrmeier A., Hedderich D.M., Dieckmeyer M., Diefenbach M.N., Ruschke S., Rummeny E.J., Zimmer C. (2019). Texture analysis of vertebral bone marrow using chemical shift encoding-based water-fat MRI: A feasibility study. Osteoporos. Int..

[28] Du J., Carl M., Bydder M., Takahashi A., Chung C.B., Bydder G.M. (2010). Qualitative and quantitative ultrashort echo time (UTE) imaging of cortical bone. J. Magn. Reson..

[29] Diefenbach M.N., Meineke J., Ruschke S., Baum T., Gersing A., Karampinos D.C. (2019). On the sensitivity of quantitative susceptibility mapping for measuring trabecular bone density. Magn. Reson. Med..

[30] Jiang H., Diao Z., Shi T., Zhou Y., Wang F., Hu W., Zhu X., Luo S., Tong G., Yao Y.D. (2023). A review of deep learning-based multiple-lesion recognition from medical images: Classification, detection and segmentation. Comput. Biol. Med..

[31] Wang P., Fan E., Wang P. (2021). Comparative analysis of image classification algorithms based on traditional machine learning and deep learning. Pattern Recognit. Lett..

[32] AlSaeed D., Omar S.F. (2022). Brain MRI analysis for Alzheimer’s disease diagnosis using CNN-based feature extraction and machine learning. Sensors.

[33] Basaia S., Agosta F., Wagner L., Canu E., Magnani G., Santangelo R., Filippi M. (2019). Alzheimer’s Disease Neuroimaging Initiative. Automated classification of Alzheimer’s disease and mild cognitive impairment using a single MRI and deep neural networks. NeuroImage Clin..

[34] Ou Yang W.Y., Lai C.C., Tsou M.T., Hwang L.C. (2021). Development of machine learning models for prediction of osteoporosis from clinical health examination data. Int. J. Environ. Res. Public Health.

[35] Wu X., Park S. (2023). A prediction model for osteoporosis risk using a machine-learning approach and its validation in a large cohort. J. Korean Med. Sci..

[36] Al Saedi A., Chen L., Phu S., Vogrin S., Miao D., Ferland G., Gaudreau P., Duque G. (2020). Age-related increases in marrow fat volumes have regional impacts on bone cell numbers and structure. Calcif. Tissue Int..

[37] Justesen J., Stenderup K., Ebbesen E.N., Mosekilde L., Steiniche T., Kassem M. (2001). Adipocyte tissue volume in bone marrow is increased with aging and in patients with osteoporosis. Biogerontology.

[38] Griffith J.F., Yeung D.K.W., Antonio G.E., Lee F.K.H., Hong A.W.L., Wong S.Y.S., Lau E.M.C., Leung P.C. (2005). Vertebral bone mineral density, marrow perfusion, and fat content in healthy men and men with osteoporosis: Dynamic contrast-enhanced MR imaging and MR spectroscopy. Radiology.

[39] Griffith J.F., Yeung D.K.W., Antonio G.E., Wong S.Y.S., Kwok T.C.Y., Woo J., Leung P.C. (2006). Vertebral marrow fat content and diffusion and perfusion indexes in women with varying bone density: MR evaluation. Radiology.

[40] Kawai M., de Paula F.J., Rosen C.J. (2012). New insights into osteoporosis: The bone-fat connection. J. Intern. Med..

[41] Gambacciani M., Ciaponi M., Cappagli B., Piaggesi L., De Simone L., Orlandi R., Genazzani A.R. (1997). Body weight, body fat distribution, and hormonal replacement therapy in early postmenopausal women. J. Clin. Endocrinol. Metab..

[42] Milisenda J.C., Collado M.V., Pinal-Fernandez I., Jaramillo A.H., Bilfeld M.F., Cano M.D., García A.I., Tomás X., Grau J.M. (2019). Correlation between quantitative and semiquantitative magnetic resonance imaging and histopathology findings in dermatomyositis. Clin. Exp. Rheumatol..

[43] Maris T.G., Damilakis J., Sideri L., Deimling M., Papadokostakis G., Papakonstantinou O., Gourtsoyiannis N. (2004). Assessment of the skeletal status by MR relaxometry techniques of the lumbar spine: Comparison with dual X-ray absorptiometry. Eur. J. Radiol..

[44] Wehrli F.W., Ford J.C., Haddad J.G. (1995). Osteoporosis: Clinical assessment with quantitative MR imaging in diagnosis. Radiology.

[45] Davis D.L., Kesler T., Gilotra M.N., Almardawi R., Hasan S.A., Gullapalli R.P., Zhuo J. (2019). Quantification of shoulder muscle intramuscular fatty infiltration on T_1_-weighted MRI: A viable alternative to the Goutallier classification system. Skelet. Radiol..

[46] Lubner M.G., Smith A.D., Sandrasegaran K., Sahani D.V., Pickhardt P.J. (2017). CT texture analysis: Definitions, applications, biologic correlates, and challenges. RadioGraphics.

[47] Fasihi L., Tartibian B., Eslami R., Fasihi H. (2022). Artificial intelligence used to diagnose osteoporosis from risk factors in clinical data and proposing sports protocols. Sci. Rep..

[48] Kruse C., Eiken P., Vestergaard P. (2017). Clinical fracture risk evaluated by hierarchical agglomerative clustering. Osteoporos. Int..

[49] Shim J.G., Kim D.W., Ryu K.H., Cho E.A., Ahn J.H., Kim J.I., Lee S.H. (2020). Application of machine learning approaches for osteoporosis risk prediction in postmenopausal women. Arch. Osteoporos..

[50] Kabir M.F., Chen T., Ludwig S.A. (2023). A performance analysis of dimensionality reduction algorithms in machine learning models for cancer prediction. Healthc. Anal..

